# Brg1 regulates murine liver regeneration by targeting miR‐187‐5p dependent on Hippo signalling pathway

**DOI:** 10.1111/jcmm.15776

**Published:** 2020-08-26

**Authors:** Junhua Gong, Tong Mou, Hao Wu, Zhongjun Wu

**Affiliations:** ^1^ Department of Hepatobiliary Surgery The First Affiliated Hospital of Chongqing Medical University Chongqing China; ^2^ Department of Hepatobiliary Surgery The Second Affiliated Hospital of Chongqing Medical University Chongqing China

**Keywords:** Brg1, Hippo signalling pathway, liver regeneration, miR‐187‐5p

## Abstract

Brg1 and Hippo signalling pathway are abnormally expressed in many malignant tumours, especially in Hepatocellular carcinoma, but their role in liver regeneration (LR) is unknown. In our research, we investigated the role of Brg1 and Hippo signalling pathway in hepatocyte proliferation and LR. Following 2/3 partial hepatectomy (PH) in liver‐specific Brg1 deleted mice (Brg1−/−) (KO) mice and sex‐matched wild‐type (WT), depletion of Brg1 in mouse embryos caused liver cell growth disorders and significantly decreased expression of miR‐187‐5p. We identified LATS1 as a target gene of miR‐187‐5p and the introduction of miR‐187‐5p decrease the expression of LATS1 and inactivated the Hippo signalling pathway, which facilitated the expression of cell cycle‐related proteins, and rescues the inhibitory effect of Brg1 in LR. Taken together, our findings suggested that deletion of Brg1 inhibits hepatocyte proliferation and LR by targeting miR‐187‐5p dependent on Hippo signalling pathway.

## 1 INTRODUCTION

The liver is one of the few tissues that can be naturally renewed. When part of the liver tissue is lost, the remnant liver tissue can regenerate into a complete liver, and the main function of liver can be restored.^1^ It is found that this is mainly due to various feedback signals that stimulate the proliferation of hepatocytes in the G0 phase; the residual hepatocytes then switch from what is basically non‐growing state to rapid growth state to proliferate and compensate for lost and damaged liver tissue and restore the normal physiological function of the liver.^2^, ^3^ This process is called liver regeneration (LR). Liver regeneration can quickly restore the main functions of the liver and maintain the balance of various physiological functions of the body.^3^ Therefore, research on the molecular mechanism of LR has become an important direction of modern medicine.

In recent years, it has been found that microRNAs (miRNAs) play important roles in the regulation of multi‐level network. Approximately half of human gene expression are regulated by miRNAs.^4^, ^5^ It has been indicated that miRNAs can play a negative or positive effect in tumour suppressor genes by down‐regulating cancer genes or tumour suppressor genes.^6^ The same results suggest that miRNAs may be an important positive or negative regulatory molecule in the process of liver regeneration.^7^, ^8^ A study found that the liver of mice with Dicer gene knocked out (blocks all miRNAs synthesis) was larger than that of normal control mice, in addition to aggravated liver damage and a gradual loss of the normal liver tissue structure.^9^ This suggests that miRNAs may participate in liver quality control and liver tissue remodelling, and the effects of negative regulatory miRNAs are stronger than those of positive regulatory miRNAs. Using miRNA arrays, Song et al identified seven miRNA expression changes in liver tissue of mice 36 hours after partial hepatectomy (PH).^10^ Shu et al found that there was a negative feedback mechanism between miRNAs expression in the early stage of LR and target maturity processing genes, which may be related to the steady‐state regulation of LR. Moreover, it was found that the up‐regulation of miRNA‐221, miRNA‐21 and the down‐regulation of miRNA‐26a promoted liver regeneration or hepatocyte proliferation.^11^ Recent studies have found that the coordinated expression of miRNAs plays an important role in human LR.^12^ However, the regulatory mechanism of liver regeneration remains to be further studied, and the role of miRNAs in LR remains unclear.

Under normal physiological conditions, there is a dynamic balance that maintains the homeostasis of the body's internal environment and the normal size of tissues and organs.^13^ When abnormal changes occur in cell proliferation or apoptosis, it is likely to transform into malignant cells, resulting in development of defects or tumour formation. In recent years, it has been found that the Hippo signalling pathway is a kinase chain composed of a series of protein kinases and transcription factors.^14^ This pathway can inhibit cell proliferation and apoptosis, regulate the dynamic balance between cell growth and death, effectively control the development of cell proliferation, organ size, the production of tumours and stabilize the internal environment.^15^ However, the regulation of hepatocyte proliferation by miRNAs and Hippo signalling pathway during LR and the molecular mechanism of these regulatory effects are not clear.

In this study, we established albumin‐Cre‐induced liver‐specific brahma‐related gene 1 (Brg1) deletion mice and found that miR‐187‐5p expression was obviously decreased in Brg1 knockout mice livers after PH; furthermore, we have studied the specific mechanism of miR‐187‐5p in hepatocytes. The results demonstrated that miR‐187‐5p could regulate hepatocyte proliferation by targeting Hippo signalling pathway. In general, Brg1 can regulate the expression of miR‐187‐5p, and miR‐187‐5p was identified as a key miRNA of hepatocyte proliferation through targeting Hippo signalling pathway in LR.

## 2 MATERIALS AND METHODS

### 2.1 Mice and PH model

To analyse the role of Brg1 in LR, we selectively deleted Brg1 in hepatocytes by mating Brg1 loxP/loxP mice with albumin‐Cre mice. Eight‐week‐old male liver‐specific Brg1 deletion mice were used for this study. We performed 2/3 partial hepatectomy (PH) on liver‐specific Brg1 deleted mice (Brg1−/−) mice (KO) and their sex‐matched wild‐type (WT). The liver and blood samples of mice were collected the designated time point for histological and serum biochemical analysis, and the corresponding nucleic acids and proteins were extracted. All animal experiments were approved by the Ethics Review Committee of the First Affiliated Hospital of Chongqing Medical University and were conducted in accordance with the Declaration of Helsinki.

### 2.2 Quantitative real‐time polymerase chain reaction (RT‐qPCR)

Total RNA was extracted from mouse liver specimens using the TRIzol reagent (TaKaRa) according to the manufacturer's protocol. miR‐4297, miR‐187‐5p, miR‐6771‐3p and miR‐8054 expression was determined by a TaqMan MicroRNA Assay kit (Applied Biosystems; Thermo Fisher Scientific, Inc). Total RNA was reverse transcribed into cDNA using PrimeScript RT Reagent (TaKaRa). RT‐qPCR was performed using SYBR Premix Ex Taq II (TaKaRa). U6 and GAPDH were used as internal references. mRNA and miRNA expression was determined using the 2^−ΔΔ^ *^C^* ^t^ method.

### 2.3 Western blot

Hepatocytes were isolated by two‐step collagenase digestion and gradient centrifugation. Total protein of hepatocytes was extracted using RIPA lysis buffer (Beyotime, People's Republic of China). A total of 15 μg of protein/well were electrophoresed by 10% sodium dodecyl sulphate polyacrylamide gel electrophoresis (SDS‐PAGE). After the protein transfer was completed, the PDVF membranes were blocked with 5% non‐fat powdered milk at room temperature for 1 hours. Next, the PDVF membranes were incubated with anti‐Brg1 (1:10 000; Abcam), anti‐CDK1 (1:10 000; Abcam), anti‐CDK4 (1:2000; Abcam), anti‐cyclin A, (1:2000; Abcam), anti‐cyclin D1, (1:10 000; Abcam), anti‐cyclin E1, (1:1000; Abcam), anti‐LATS1, (1:5000; Abcam), anti‐p‐YAP, (1:5000; Abcam), anti‐YAP, (1:5000; Abcam) and anti‐GAPDH antibody (1:1000, Abcam) at 4°C overnight. GAPDH was used as an internal reference. Finally, enhanced chemiluminescence (ECL) (Thermo Fisher) was used to detect the expression of the target proteins.

### 2.4 Reintroduction of miR‐187‐5p agomir in mice liver

The miR‐187‐5p agomir (5 nmol/mouse) or an miRNA negative control (Ribo‐bio, People's Republic of China) was injected via tail vein into Brg1−/− mice (n = 20) at 6 hours after 2/3 PH, and liver samples were collected at the designed experimental time points.

### 2.5 Histology and immunohistochemical staining

We used haematoxylin‐eosin (HE) staining and immunohistochemical staining to assess the pathological changes and the expression of Brg1, BrdU and Ki‐67, respectively, in paraffin‐embedded sections of mouse livers. For Brg1, BrdU and Ki‐67 immunohistochemistry staining, the slices were incubated with anti‐Brg1 (1:200; Abcam), anti‐BrdU (1:400; Abcam) and anti‐Ki‐67 (1:500; Abcam) overnight at 4°C, followed by biotinylated secondary antibody at 37°C for 1 hour. Colour development was carried out with DAB (3,3‐diaminobenzidine) and running water for 5 minutes. The slides were counterstained with 1% Mayer's haematoxylin. Brg1, BrdU and Ki‐67 immunostaining were scored and examined by two independent assessors. KO group and WT group were used for immunohistochemical detection (n = 5).

### 2.6 Luciferase reporter assay

A luciferase reporter assay was used to whether miR‐187‐5p can directly regulate LATS1. The wild‐type LATS1‐3′UTR (WT) and mutant LATS1‐3′UTR (MUT) containing the putative binding site of miR‐187‐5p were amplified by GenePharma (Shanghai, People's Republic of China) and cloned into the firefly luciferase‐expressing pMIR‐REPORT vector (Obio Technology, People's Republic of China). The luciferase reporter vector and miR‐187‐5p mimic, miR‐187‐5p inhibitor and miR‐NC were transiently co‐transfected using Lipofectamine 2000. Luciferase assays were performed using the Luciferase Reporter Assay System (GloMax).

### 2.7 miRNA target prediction

Two prediction databases, including TargetScan (http://www.targetscan.org) and miRWalk (http://mirwalk.umm.uni‐heidelberg.de/)were used to predict miRNA targets and conserved sites bound by Brg1.

### 2.8 Statistical analysis

GraphPad Prism version 6.0 or SPSS 20.0 software was used for statistical analysis. All data are presented as the means ± standard deviation (SD). Statistical differences were analysed by Student's *t* test, while the significance of differences between multiple groups was determined by one‐way analysis of variance, followed by the Newman‐Keuls test, and repeated measures analysis of variance. *P*‐value < .05 indicated statistically significant.

## 3 RESULTS

### 3.1 The construction of hepatocyte‐specific Brg1‐deficient mouse

**FIGURE 1:**
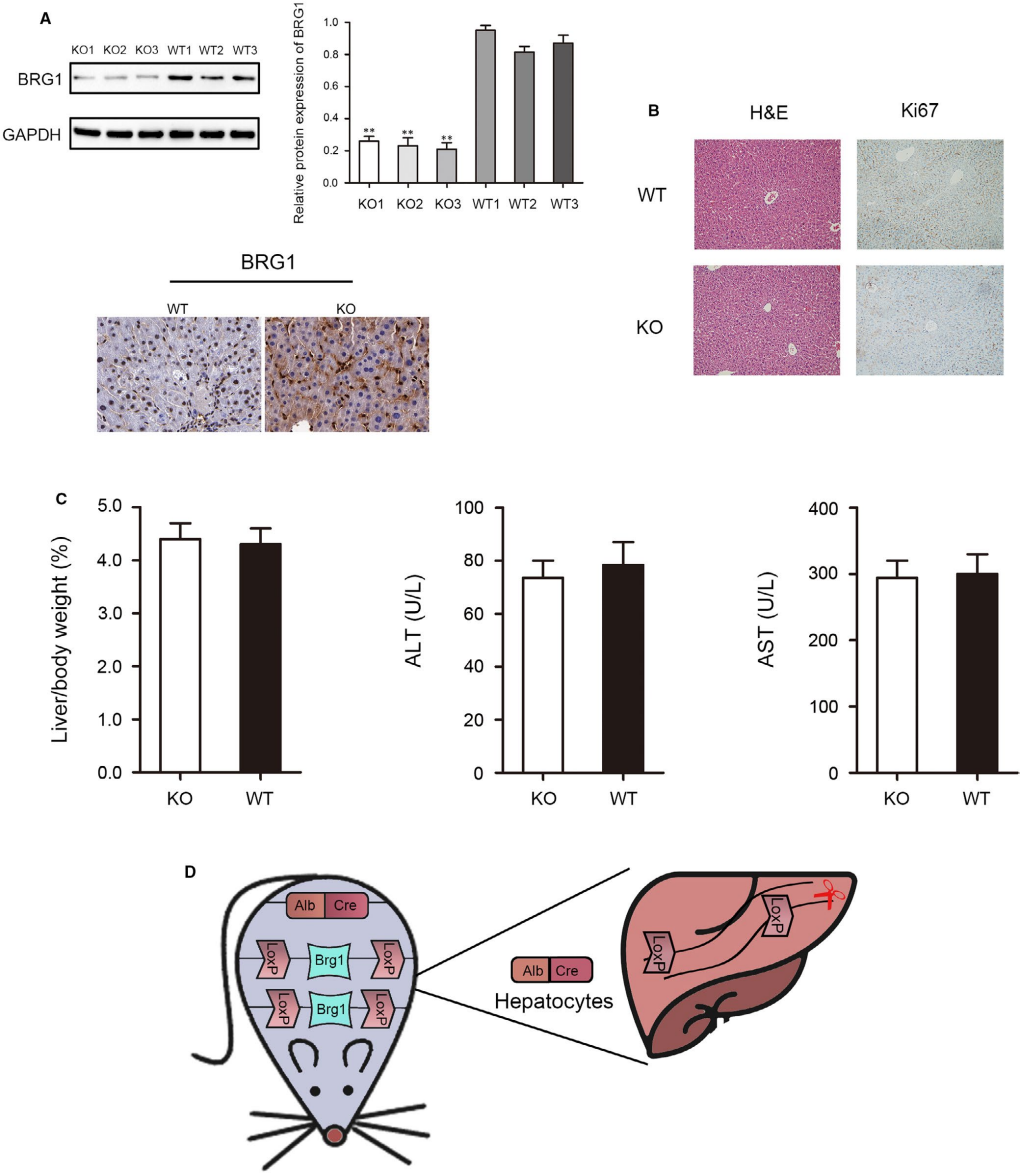
The construction of hepatocyte‐specific Brg1‐deficient mice. A, Western blot (WB detects the protein extracted from hepatocytes) and immunohistochemistry were used to test the expression of Brg1 in KO and WT mice. B, HE and Ki67 staining to test the liver condition of KO and WT mice. C, The serological detection and liver/ bodyweight ratio results from KO and WT mice. D, Schematic diagram of the construction of hepatocyte‐specific Brg1‐deficient mice. All data are represented as the mean ± SD, **P < .01

To assess the role of Brg1 in LR, we selectively deleted Brg1 in hepatocytes by mating Brg1 loxP/loxP mice with albumin‐Cre mice. Western blot and immunohistochemistry were used to check the Brg1 knockout efficiency in the liver, and the results show that the expression of Brg1 in KO mice was significantly lower than that in WT mice (Figure 1A). The results of HE and Ki‐67 showed no significant damage to the liver in KO or WT mice (Figure 1B). Moreover, serological detection and liver/weight ratio results showed no significant difference in KO and WT mice (Figure 1C). These results showed that the acute depletion of Brg1 did not cause obvious liver injury. Shown in Figure 1D is a schematic diagram of the specific knockout of Brg1 in the liver.

### 3.2 Acute depletion of Brg1 significantly inhibited liver regeneration in mice

**FIGURE 2:**
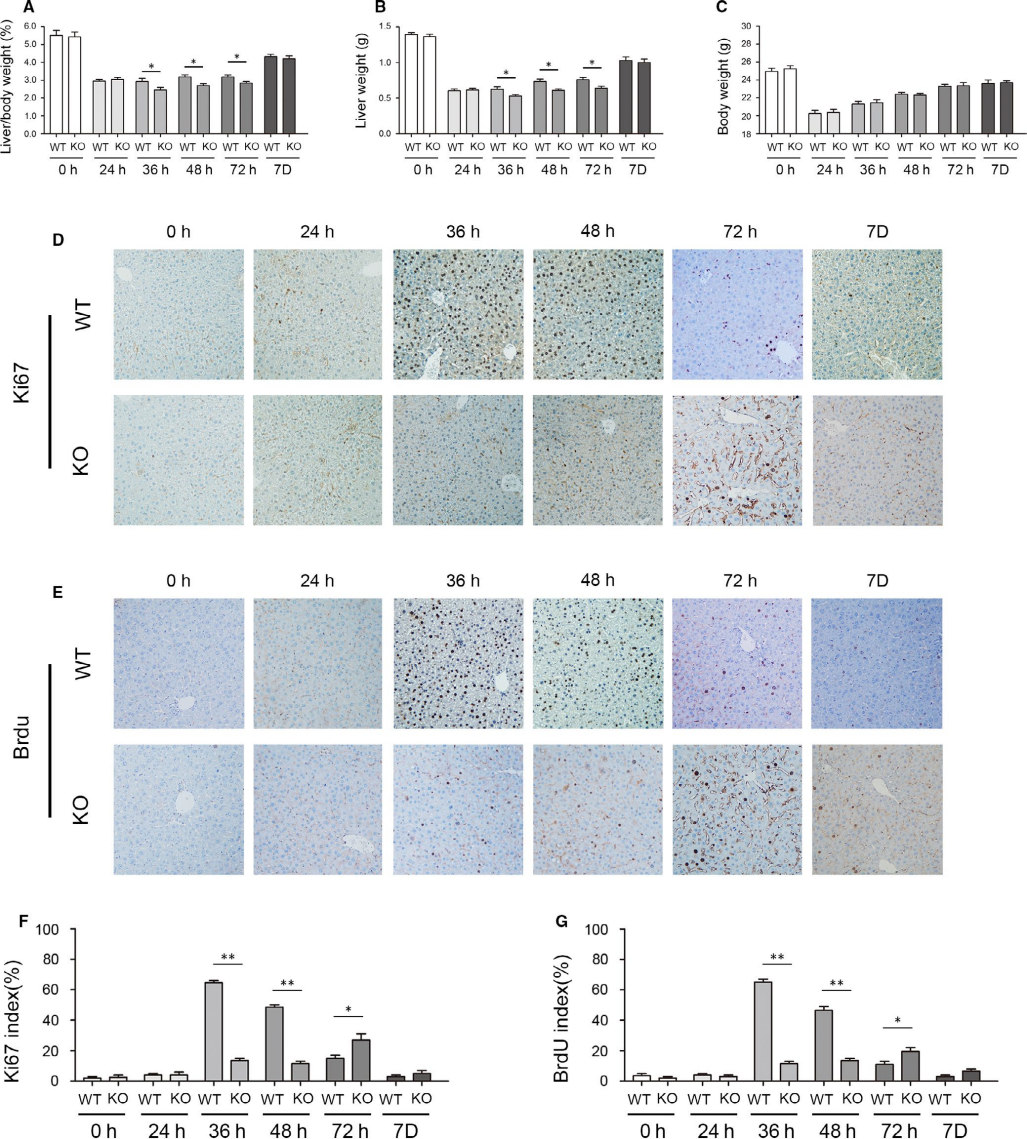
Liver regeneration is impaired in KO and WT mice. A, The liver/bodyweight ratio results at indicated time points after 2/3 PH in KO and WT mice. B, The liver weight results at indicated time points after 2/3 PH in KO and WT mice. C, The bodyweight results at indicated time points after 2/3 PH in KO and WT mice. D, Ki67 staining of liver sections at indicated time points after 2/3 PH in KO and WT mice. E, BrdU staining of liver sections at indicated time points after 2/3 PH in KO and WT mice. F, The statistic results of Ki67‐positive cell ratios at indicated time points after 2/3 PH in KO and WT mice (in a field of view at 200 x magnification). G, The statistic results of BrdU‐positive cell ratios at indicated time points after 2/3 PH in KO and WT mice (in a field of view at 200 x magnification). All data are represented as the mean ± SD, *P < .05, **P < .01

We performed classic 2/3 PH in KO and WT mice to determine the role of Brg1 in liver regeneration. After the operation, the remnant liver was sampled at 0, 24, 36, 48, 72 hours, 5 and 7 day time points for detection. In the early stage of liver regeneration (36, 48 and 72 hours), the liver/body weight ratio of KO mice were lower than that of WT mice (Figure 2A). In addition, both KO and WT mice could complete liver regeneration within 7 days (Figure 1B, C). To study the mechanism of Brg1 on liver regeneration in mice, we detected the expression of Ki‐67 and BrdU in at each time point during liver regeneration (Figure 1D, E). At 36 and 48 hours after PH, the number of Ki‐67‐positive cells in KO mice (17.67% and 16.34%) was significantly lower than that in WT mice (69.23% and 57.07%) (Figure 1F). Moreover, the number of Ki‐67‐positive cells in KO mice was obviously higher than that in WT mice at 72 hours after PH (Figure 1F). In addition, at 36 and 48 hours after PH, the number of cells positive for BrdU in KO mice (16.32% and 17.09%) was significantly lower than that in WT mice (70.23% and 52.31%) (Figure 1G). Moreover, the number of cells positive for BrdU in KO mice was obviously higher than that in WT mice at 72 hours after PH (Figure 1G).

### 3.3 Brg1 affects liver regeneration in mice by regulating Hippo signalling pathway through miR‐187‐5p

**FIGURE 3:**
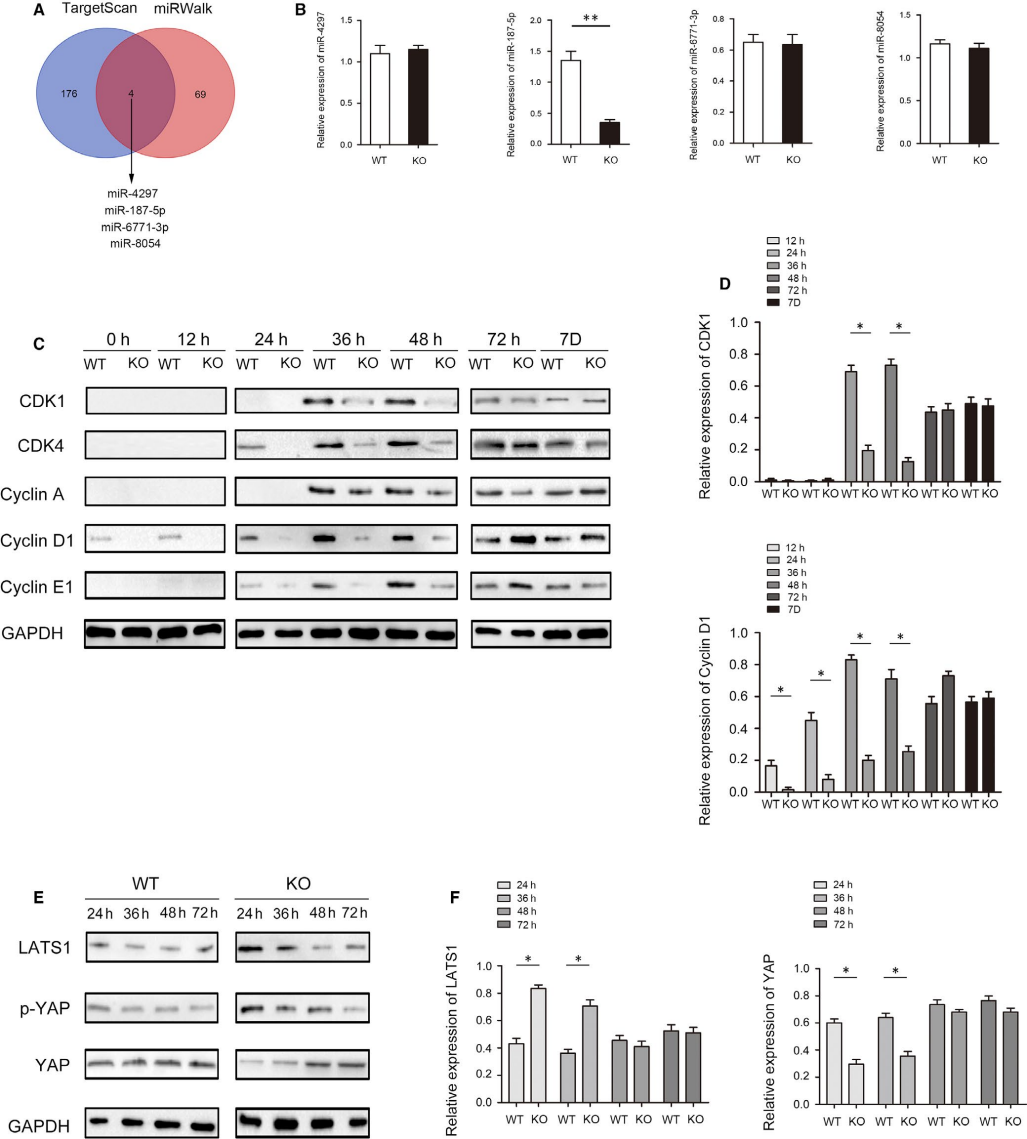
Detection and quantification of miR‐187‐5p, cyclins, CDKs and key regulator proteins of hippo signalling pathway in regenerated liver. A, Venn diagram displaying Brg1 target miRNAs by TargetScan and miRWalk. B, RT‐qPCR was used to test the expression of miR‐4297, miR‐187‐5p, miR‐6771‐3p and miR‐8054 in KO mice and WT mice. C, The CDK1, CDK4, cyclin D1, cyclin A and cyclin E1 at indicated time points after 2/3 PH in KO and WT mice. D, The statistic results of CDK1 and cyclin D1 at indicated time points after 2/3 PH in KO and WT mice. E, The protein of LATS1, p‐YAP and YAP at indicated time points after 2/3 PH in KO and WT mice. F, The statistic results of LATS1 and YAP at indicated time points after 2/3 PH in KO and WT mice. All data are represented as the mean ± SD, *P < .05, **P < .01

Considering that the biological function of Brg1 also involves the formation and maturity of miRNAs, we conducted a predictive analysis of miRNAs correlations using online database, including TargetScan (http:// www.targetscan.org) and miRWalk (http://mirwalk.umm.uni‐heidelberg.de/). The results show that miR‐4297, miR‐187‐5p, miR‐6771‐3p and miR‐8054 were potential target gene of Brg1 (Figure 3A). In addition, the RT‐qPCR results show that the expression of miR‐187‐5p in KO mice was significantly lower than that in WT mice, and however, there was no significant difference in the expression of miR‐4297, miR‐6771‐3p and miR‐8054 (Figure 3B). When analysing the expression of several cyclins and CDKs in KO and WT mice, we found that CDK1, CDK4, cyclin D1, cyclin A and cyclin E1 were all lower in KO compared to WT mice before 72 hours after PH (Figure 3C, D).

There is much evidence that Hippo signalling pathway is involved in the growth and development of tissues and organs. YAP, phosphorylated by LATS1, can inactivate Hippo signalling pathway, which is pivotal for the expression of cyclins and CDKs. Therefore, a new research direction for us to study why the absence of Brg1 causes a delay in liver regeneration. We use Western blot to detect the expression of LATS1, p‐YAP and YAP in the process of liver regeneration at 24, 36, 48 and 72 hours. The results show that LATS1 and p‐YAP were highly expressed in KO mouse livers, while YAP was lower after 70% PH (Figure 3E, F).

### 3.4 miR‐187‐5p rescues the Brg1‐induced delay in liver regeneration

**FIGURE 4:**
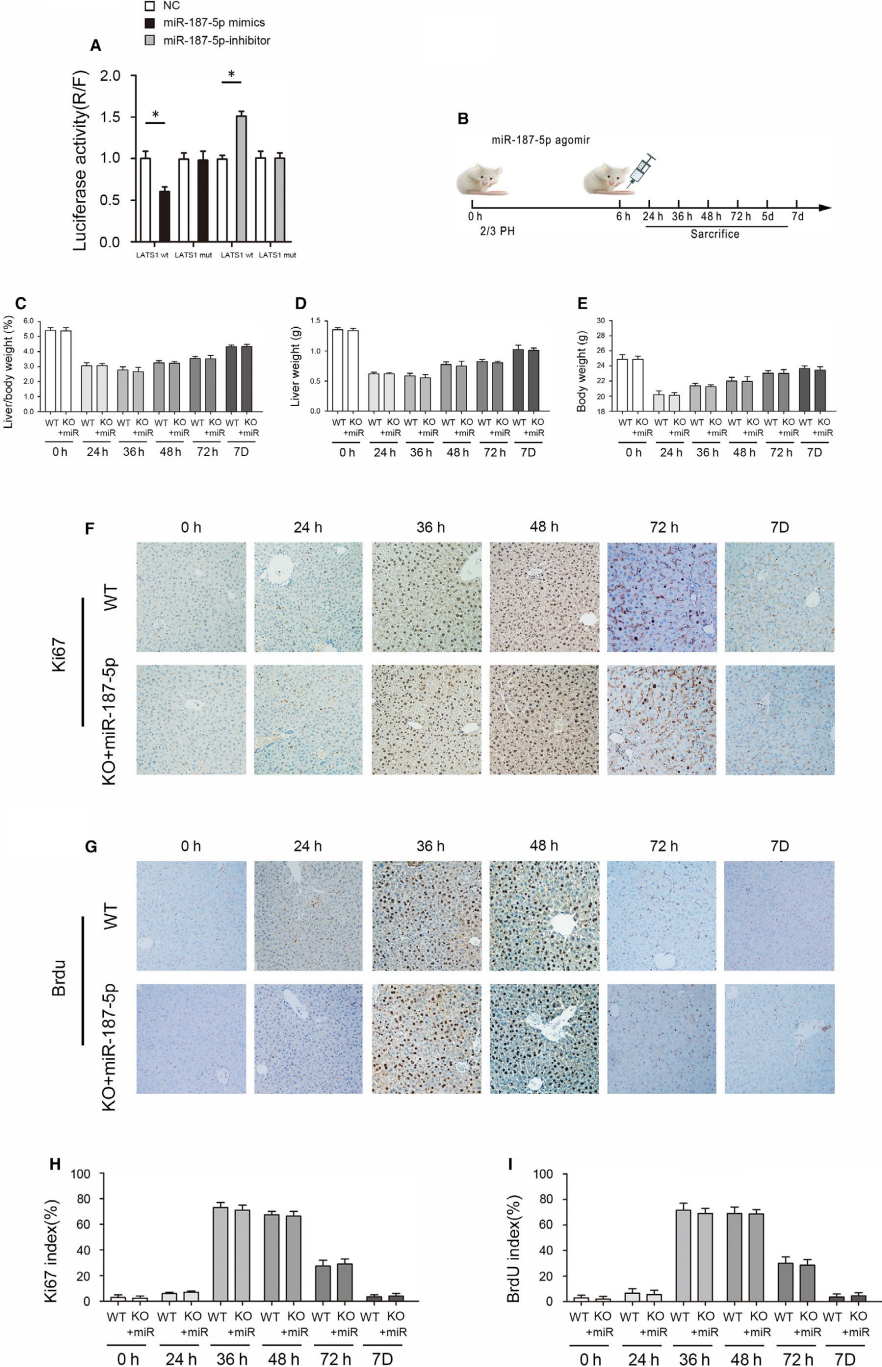
miR‐187‐5p rescues the inhibition effect of liver regeneration. A, Luciferase activity was used to detect the direct effect of miR‐187‐5p and LATS1. B, Schematic diagram for tail vein injection with miR‐187‐5p agomir. C, The liver/ bodyweight ratio results at indicated time points after 2/3 PH in KO and WT mice. D, The liver weight results at indicated time points after 2/3 PH in KO and WT mice. E, The bodyweight results at indicated time points after 2/3 PH in KO and WT mice. F, Ki67 staining of liver sections at indicated time points after 2/3 PH in KO and WT mice. G, BrdU staining of liver sections at indicated time points after 2/3 PH in KO and WT mice. H, The statistic results of Ki67‐positive cell ratios at indicated time points after 2/3 PH in KO and WT mice (in a field of view at 200× magnification). I, The statistic results of BrdU‐positive cell ratios at indicated time points after 2/3 PH in KO mice and WT mice (in a field of view at 200× magnification). *P < .05

The Luciferase reporter assay results show that co‐transfection of miR‐187‐5p inhibitor significantly increased luciferase activity in cells transfected with WT LATS1 3′‐UTR. In contrast, there was no growth phenomenon in cells co‐transfected with MUT LATS1 3′‐UTR (Figure 4A). In addition, we injected KO mice with the miR‐187‐5p agomir via tail vein at 6 hours after 2/3 PH, and the remnant liver was sampled at 0, 24, 36, 48, 72 hours, 5 and 7 day time points for detection (Figure 4B). The results showed that the delay of liver regeneration caused by the acute absence of Brg1 was significantly relieved (Figure 4C‐E). Moreover, compared with WT mice, the expression of CDK1, CDK4, cyclin D1, cyclin A and cyclin E1 in KO mice was significantly restored (Figure 4E‐I).

### 3.5 miR‐187‐5p restores Hippo signal pathway in liver regeneration

**FIGURE 5:**
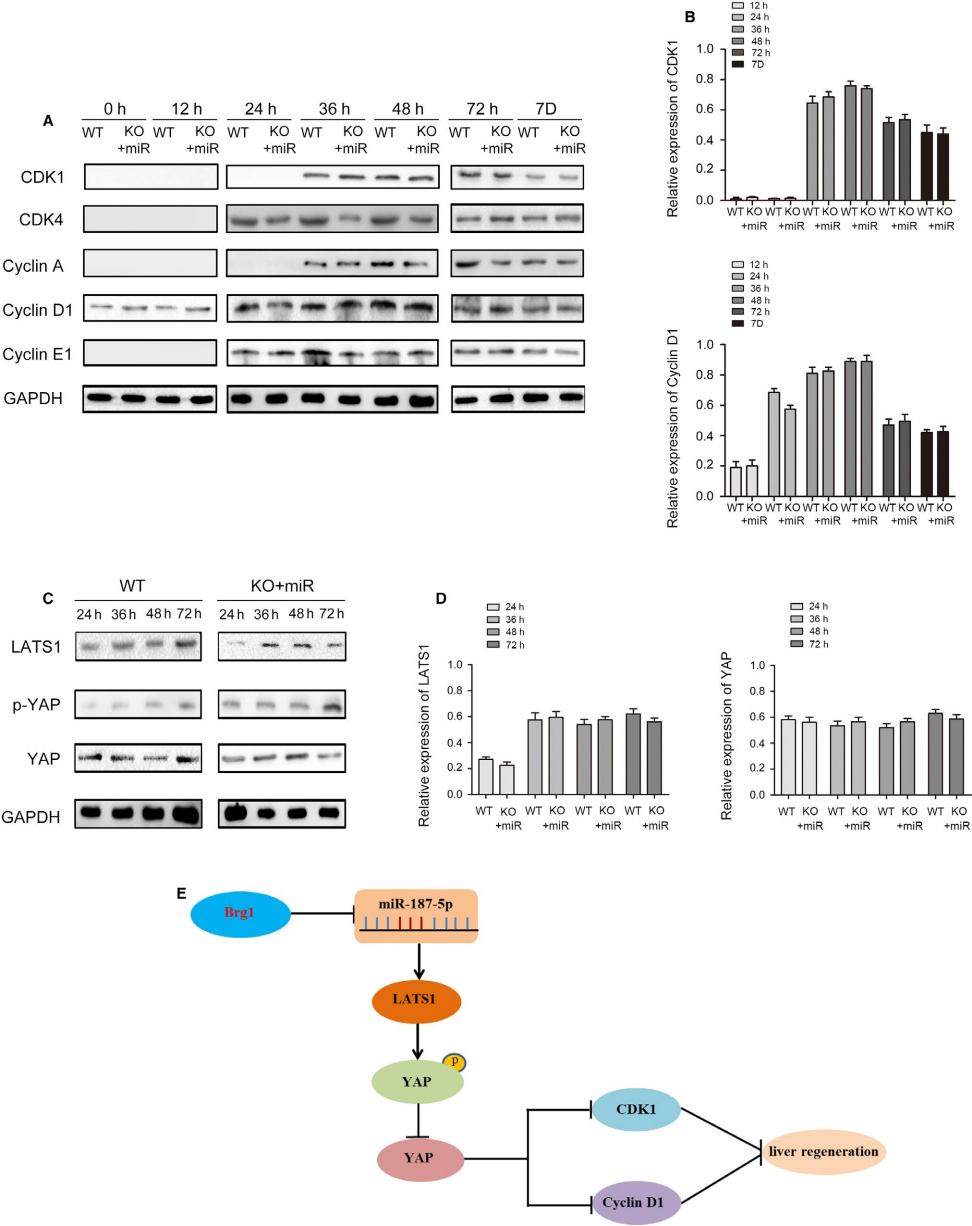
Detection and quantification of cyclins, CDKs and key regulator proteins of hippo signalling pathway in regenerated liver. A, The CDK1, CDK4, cyclin D1, cyclin A and cyclin E1 at indicated time points after 2/3 PH in KO and WT mice. B, The statistic results of CDK1 and cyclin D1 at indicated time points after 2/3 PH in KO and WT mice. C, The LATS1, p‐YAP and YAP at indicated time points after 2/3 PH in KO and WT mice. D, The statistic results of LATS1 and YAP at indicated time points after 2/3 PH in KO and WT mice. E, Proposed mechanism of Brg1 affects liver regeneration in mice by regulating Hippo signalling pathway through miR‐187‐5p

In addition, the expression level of cyclins, CDKs and the key proteins of Hippo signal pathway (LATS1, p‐YAP and YAP) was analysed by Western blot. The results showed that the expression disparity of cyclins, CDKs, LATS1, p‐YAP and YAP was almost eliminated after miR‐187‐5p agomir transfusion (Figure 5A‐D). Based on our findings, we confirmed that Brg1 regulates murine liver regeneration by targeting miR‐187‐5p dependent on Hippo signalling pathway (Figure 5E).

## 4 DISCUSSION

A large number of studies show that miRNA not only affect individual development, cell metabolism, apoptosis, ageing, immune response, malignant tumour and other life processes but also participate in complex pathophysiological processes of various systems of human body, especially liver regeneration.^16^, ^17^ Furthermore, Brg1 is the catalytic subunit of the SWItch/Sucrose Non‐Fermentable (SWI/SNF) complex.^18^ Based on the above results, we constructed liver‐specific Brg1 deletion mice, performed a classical 2/3 partial hepatectomy and observed changes in miRNA and liver regeneration‐related indicators after surgery. The role of Brg1 in LR has been reported, but the specific mechanism of Brg1 in LR from the perspective of miRNAs has not been reported. Therefore, our experimental model provides scientific and objective exploration for studying the molecular mechanism of Brg1 and miRNA in liver regeneration.

miR‐187‐5p is a miRNA that plays an important role in the physiological and pathological process of the body.^19^ It was found that the abnormal expression of miR‐187‐5p was closely related to paclitaxel and docetaxel drug resistance in breast cancer. Moreover, The G2‐M checkpoint of cell cycle is the target of paclitaxel in breast cancer, and miR‐187‐5p can significantly regulate G2‐M checkpoint of cell cycle.^20^ In bladder cancer, high expression of miR‐187‐5p is closely related to the poor prognosis of bladder cancer patients. Inhibition of miR‐187‐5p expression can significantly reduce the occurrence of malignant biological behaviour of bladder cancer.^21^ In addition, miR‐187‐5p is a tumour suppressor miRNA in non‐small lung cancer progression, overexpression of miR‐187‐5p can significantly inhibit the expression of CYP1B1 and reduce the growth and metastasis of non‐small lung cancer.^22^ Importantly, miR‐187‐5p/Apaf‐1 axis participates in oxidative stress‐mediated apoptosis of liver through mitochondrial pathway.^23^ In our existing studies, we found that during liver regeneration in liver‐specific Brg1 deletion mice, the cyclin expression of cell proliferation can be influenced by miR‐187‐5p, and then, the process of liver regeneration can be regulated.

Brahma‐related gene 1 (Brg1) is the core component of SWI/ SNF chromatin remodelling complex in mammals and has ATPase activity.^24^ Brg1 plays a key role in the pathogenesis of various human diseases by shaping the landscape of gene expression. Conditional knockout of Brg1 in hepatocytes reduced the expression of galectin‐3 in different liver injury models. Further analysis showed that Brg1 interacted with AP‐1, bound to promoter of proximal galectin‐3 and activated transcription.^25^ At the same time, Brg1 recruited DNA 5‐methylcytosine dioxygenase TET1 to galectin‐3 to promote the demethylation of active DNA and activate galectin‐3 transcription. Finally, Tet1 silencing blocked LPS and palmitate induced galectin‐3 expression in hepatocytes.^25^ In addition, Brg1 interacts with AP‐1 and SMAD3 and mediates TGF ‐ β‐induced transcription of NOX4 in endothelial cells.^25^ By mechanism, Brg1 recruits a variety of histone‐modifying enzymes to change the chromatin structure around the NOX4 site, thus activating its transcription and stimulating liver fibrosis.^26^ In addition, the absence of Brg1 in endothelial cells resulted in the up‐regulation of eNOS activity and an increase in NO bioavailability, which also led to the remission of liver fibrosis. By mechanism, BRG1 interacts with MLL1 to regulate H3K4 trimethylation around CAV1 promoter, thus promoting CAV1 activation by LPS.^27^ In liver fibrosis, Brg1 regulates hepatic stellate cells activation via TGF β/SMAD signal pathway. In BRG1‐deficient mice, the development and occurrence of liver fibrosis decreased after CCl4 chronic injury. In addition, the expression of BRG1 was positively correlated with liver fibrosis in patients with cirrhosis, which may be a prognostic factor for liver cancer.^28^ Our results are also consistent with previous studies. The absence of Brg1 resulted in a decrease in miR‐187‐5p expression, which significantly delays the process of liver regeneration. These results showed that Brg1 plays a key role in the physiological and pathological process of liver.

miRNAs perform their biological functions through their target genes.^29^, ^30^ Many studies have shown that the role of miRNAs in LR proliferation depends on their specific target genes.^31^, ^32^, ^33^ Therefore, we focused on the relevant signalling pathways, that regulate cell growth and proliferation. The Hippo signalling pathway is a highly conserved signalling pathway that can regulate animal cell division, organ size and tumourigenesis; it was identified in studies of Drosophila melanogaster growth and development.^34^ As the core of hippo signalling pathway, large tumour suppressor gene 1 (LATS1) can phosphorylate Yes‐associated protein (YAP), and phosphorylated YAP interacts with 14‐3‐3 protein.^35^ The interaction between phosphorylated YAP and 14‐3‐3‐ protein sequesters YAP in the cytoplasm, thereby blocking its entry into the nucleus and inhibiting its transcriptional function. When YAP is not phosphorylated, it can enter the nucleus as a non‐DNA‐binding auxiliary transcription factor; there it can bind to the transcription factor TEAD and then the binding region of the YAP/TEAD complex binds to DNA and induces downstream target gene transcription. YAP exerts transcriptional co‐activation to regulate cell proliferation‐related genes and tumour apoptosis inhibitory genes, thereby breaking the dynamic balance of cell growth and benefiting tumour proliferation and metastasis.^36^, ^37^, ^38^ Our results indicate that the reduced expression of miR‐187‐5p during LR in liver‐specific Brg1‐deleted mice caused activation of the Hippo signalling pathway via increased LATS1, resulting in decreased expression of cell cycle‐related proteins and delayed liver regeneration. This major disparity was compensated by transfusing miR‐187‐5p analogues, which could inactivate the Hippo signalling pathway and increased expression of cell cycle‐related proteins after depletion of Brg1 such that hepatocytes can enter into cell cycle and the inhibitory effect of Brg1 deletion on LR.

## 5 CONCLUSIONS

Liver‐specific Brg1 deleted leads to liver regeneration. Moreover, Brg1 deletion causes Brg1‐dependent miR‐187‐5p reduction, and activation of the Hippo signalling pathway via increased LATS1. The introduction of miR‐187‐5p decreased the expression of LATS1 and inactivated of the Hippo signalling pathway, which facilitates the expression of cell cycle‐related proteins and rescued the inhibition effect of Brg1 in LR.

## CONFLICT OF INTEREST

None of the authors has a conflict of interest statement in relation to this article.

## AUTHOR CONTRIBUTIONS

**Junhua Gong:** Data curation (equal); Investigation (equal); Methodology (equal); Project administration (equal); Resources (equal); Software (equal); Supervision (equal); Validation (equal); Visualization (equal); Writing‐original draft (equal); Writing‐review & editing (equal). **Tong Mou:** Conceptualization (equal); Data curation (equal); Resources (equal); Software (equal); Supervision (equal); Validation (equal). **Hao Wu:** Conceptualization (equal); Data curation (equal); Formal analysis (equal); Investigation (equal); Methodology (equal); Project administration (equal); Resources (equal); Software (equal); Supervision (equal); Validation (equal); Visualization (equal); Writing‐original draft (equal); Writing‐review & editing (equal). **Zhongjun Wu:** Conceptualization (equal); Data curation (equal); Formal analysis (equal); Funding acquisition (equal); Investigation (equal); Methodology (equal); Project administration (equal); Resources (equal); Software (equal); Supervision (equal); Validation (equal); Visualization (equal); Writing‐original draft (equal); Writing‐review & editing (equal).

## Data Availability

The data used to support the findings of this study are available from the corresponding author upon request.
